# Combined Magnetomotive ultrasound, PET/CT, and MR imaging of ^68^Ga-labelled superparamagnetic iron oxide nanoparticles in rat sentinel lymph nodes *in vivo*

**DOI:** 10.1038/s41598-017-04396-z

**Published:** 2017-07-06

**Authors:** Maria Evertsson, Pontus Kjellman, Magnus Cinthio, Roger Andersson, Thuy A Tran, Rene in’t Zandt, Gustav Grafström, Hanna Toftevall, Sarah Fredriksson, Christian Ingvar, Sven-Erik Strand, Tomas Jansson

**Affiliations:** 1 Department of Biomedical Engineering, Faculty of Engineering LTH at Lund University, Lund, Sweden; 2 Lund University, Faculty of Medicine, Department of Clinical Sciences Lund, Medical Radiation Physics, Lund, Sweden; 3 Medical Services, Skåne University Hospital, Lund, Sweden; 4 Lund University Bioimaging Center, Lund University, Lund, Sweden; 5 Lund University, Faculty of Medicine, Department of Clinical Sciences Lund, Division of Oncology-Pathology, Lund, Sweden; 6 Genovis AB, Lund, Sweden; 7 Department of Surgery, Skåne University Hospital, Lund, Sweden; 8 Lund University, Faculty of Medicine, Department of Clinical Sciences Lund, Biomedical Engineering, Lund, Sweden

## Abstract

Current methods for intra-surgical guidance to localize metastases at cancer surgery are based on radioactive tracers that cause logistical challenges. We propose the use of a novel ultrasound-based method, magnetomotive ultrasound (MMUS) imaging that employ a nanoparticle-based contrast agent that also may be used for pre-operative PET/MRI imaging. Since MMUS is radiation free, this eliminates the dependence between pre- and intra-operative imaging and the radiation exposure for the surgical staff. This study investigates a hypothetical clinical scenario of pre-operative PET imaging, combined with intra-operative MMUS imaging, implemented in a sentinel lymph node (SLN) rat model. At one-hour post injection of ^68^Ga-labelled magnetic nanoparticles, six animals were imaged with combined PET/CT. After two or four days, the same animals were imaged with MMUS. In addition, *ex-vivo* MRI was used to evaluate the amount of nanoparticles in each single SLN. All SLNs were detectable by PET. Four out of six SLNs could be detected with MMUS, and for these MMUS and MRI measurements were in close agreement. The MRI measurements revealed that the two SLNs undetectable with MMUS contained the lowest nanoparticle concentrations. This study shows that MMUS can complement standard pre-operative imaging by providing bedside real-time images with high spatial resolution.

## Introduction

Non-invasive imaging for cancer staging is currently mostly conducted using nuclear imaging methods such as single-photon emission computed tomography (SPECT) and positron emission tomography (PET), both of which are sensitive methods that provide whole-body imaging with a spatial resolution in the order of mm^1, 2^. The two modalities provide functional imaging, that enables localization and quantification of activity, but do not reveal anatomical details. For morphological imaging with high spatial resolution, computed tomography (CT), magnetic resonance imaging (MRI)^3–7^, and ultrasound^4, 5, 8^ can be added. Hybrid systems such as SPECT/CT, PET/CT, and PET/MRI have been developed and are now in clinical use^9–11^. As these systems provide images that combine high sensitivity with detailed anatomical information, they are excellent tools for pre-operative staging.

In the case of exploring potential distal metastases in lymph nodes during surgery, however, other imaging methods must be used. For common cancers as breast cancer and malignant melanoma where the majority of patients are treated using the sentinel node biopsy technique, the gold standard is to inject contrast agents in the form of radioactive nanocolloids and blue dye prior to surgery. These contrast agents are injected peritumorally or intradermally and accumulate in the sentinel lymph node (SLN), the first lymph node receiving lymphatic drainage from the primary tumor and therefore the most likely location for regional disease. Consequently, it is of utmost importance to localize and histologically examine the SLN as regional disease still is considered the most important prognostic factor. Intra-operatively the practice is to locate the SLN using a handheld gamma probe, as a guide, and the blue dye to visually help the surgeon identify the SLN during resection^12–15^. Since the radioactive tracer is used for SLN detection both pre-operatively and intra-operatively, the radioisotope’s half-life and the injected radioactive dose must be sufficient to enable SLN detection in both procedures. On the other hand, the total radioactivity should be as low as possible to minimize the absorbed dose. Several isotopes have been studied but ^99m^Tc is currently used in most centers. With a half-life of six hours for ^99m^Tc the timing of pre-operative imaging and the need of a located uptake in the SLN is a logistic challenge. A further complication with this procedure is a risk of allergic reactions associated with the blue dye^14, 15^.

To eliminate the dependence between pre- and intra-operative imaging, we propose to use radiolabelled superparamagnetic iron oxide nanoparticles (SPIONs) as a multimodal contrast agent. This allows for pre-operative staging with PET followed by intra-operative detection of the particles using a developing ultrasound based modality called magnetomotive ultrasound imaging (MMUS), which does not employ radioactivity for contrast agent localization.

Ultrasound is currently the most commonly used imaging technique in health care. The technique has several advantages over other methods in that it is cost-effective, non-invasive, non-ionizing, portable, and it provides real-time images with both anatomical and physiological information. Imaging of SPIONs with conventional ultrasound is not possible, however, because the particles are too small to backscatter ultrasound at a detectable level^5, 8, 16, 17^. To overcome this limitation, MMUS indirectly image the nanoparticles by virtue of an external time-varying magnetic field that set SPIONs in motion. In turn, the tissue associated with the nanoparticles will be set in motion, and this movement (i.e. magnetomotive movement) can be detected with ultrasound shown in both phantoms^16–21^ and animals^22–25^. In addition, the MMUS technique has also been combined with photoacoustic imaging^26, 27^. Owing to the much smaller size of SPIONs as compared to conventional ultrasound contrast agents, the MMUS technique has the potential for numerous new applications in the medical imaging field by enabling real-time imaging of molecular events^17^.

In this study, we aim to demonstrate the usefulness of MMUS in an animal model (rat) mimicking a hypothetical clinical SLN work flow in which PET, MRI and MMUS are combined using ^68^Ga-labelled SPIONs as a multimodal contrast agent. We envision that highly sensitive and quantitative PET imaging together with high-resolution anatomical MRI are used for pre-operative imaging and then, as an intra-operative guide, MMUS for visualization of the SLN, utilizing the very same particles for all modalities. Since the SPION retention time in the SLN is long, together with the fact that the MMUS signal is only dependent on the concentration of the SPIONs, intra-operative SLN detection can be performed several days after nanoparticle administration when the radioactivity has decayed. This carries logistical advantages as compared to current clinical procedure. Additionally, a radionuclide with a shorter half-life can be used, which is also demonstrated in this study with ^68^Ga (half-life of 68 minutes). This results in a low absorbed dose to the patient and an elimination of radiation exposure to the surgical staff. Thus, our suggested technology shift has the potential for more rational handling of SLN resection. Further, the blue dye and the complications thereof are also eliminated.

The aim with the present animal study was to test this hypothetical clinical scenario of multimodal imaging where PET/CT images were acquired one hour after nanoparticle injection followed by MMUS imaging either two or four days later. In this experiment, MRI was used to validate the presence of SPIONs.

## Results

### PET/CT imaging

**Figure 1:**
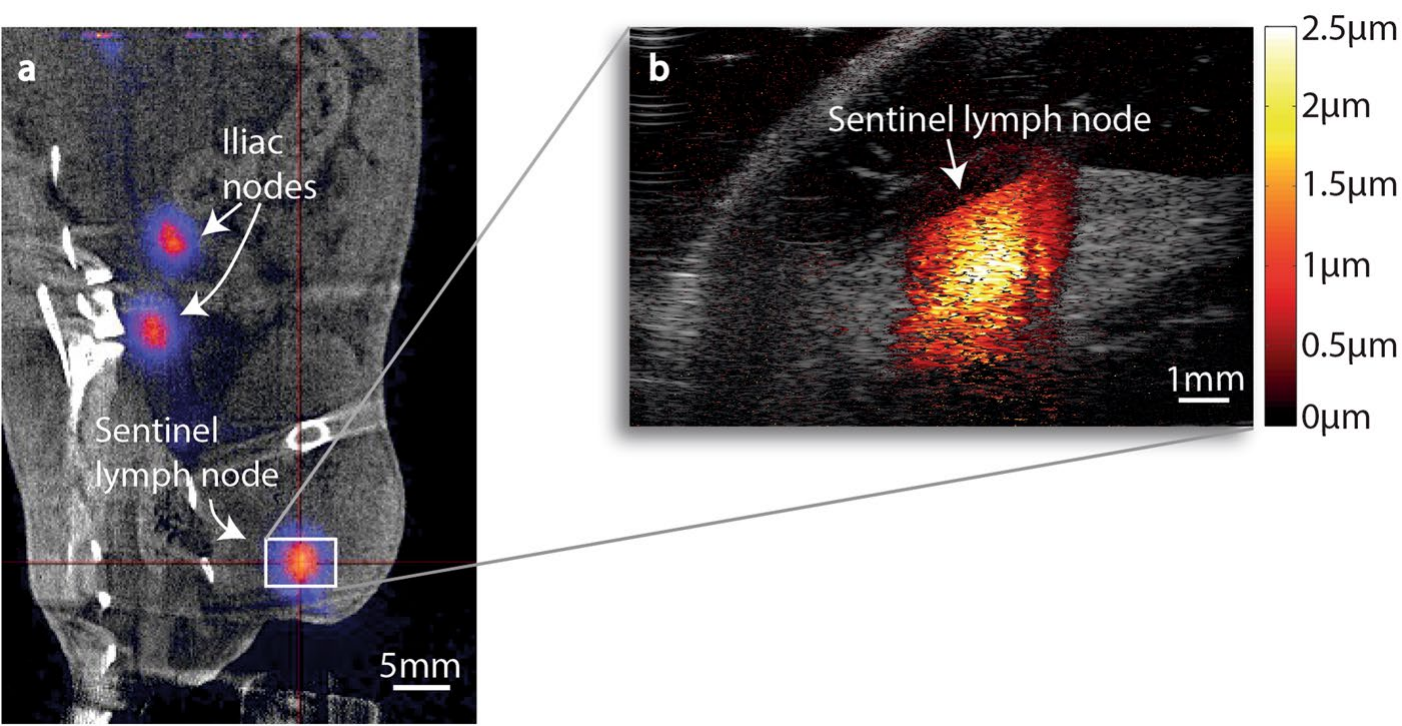
(**a**) Coronal PET/CT image of animal 1. The centre of the red cross shows the position of the nanoparticle-accumulated sentinel lymph node. Nanoparticle accumulation is also seen in two iliac lymph nodes. The lead cylinder shielding the injected paw is shown to the bottom right. (**b**) MMUS image of the same sentinel lymph node. The image is a magnification of the area outlined with a white rectangle in the PET/CT image. The induced magnetomotive displacement is colour-coded in the image according to the colour-bar to the right.

To test the hypothetical scenario we used six Wistar rats injected, in their right hind paw, with polyethylene glycol coated SPIONs (iron oxide core diameter 10 ± 2 nm, hydrodynamic diameter to 40 ± 5 nm as determined by dynamic light scattering) labelled with ^68^Ga. In this model, the popliteal lymph node (in the knee) served as the sentinel lymph node. One hour post injection a PET/CT scan of each animal was performed where ^68^Ga-labelled SPIONs were successfully detected in all six animals. As an example, a coronal PET/CT image is shown in Fig. 1A. The uptake of radioactivity was highest in the sentinel lymph node in all animals but radioactivity was also found in the second and sometimes also the third lymph node (iliac nodes).

The mean percent injected activity (%IA) (± standard deviation) in the SLNs as determined from the 45-minute PET scans for all six animals was 2.6 ± 2.9%, whereas the mean %IA in the contralateral lymph node was 0.1 ± 0.05%, which was considered as background. Individual data for each animal is provided in Table 1.

**Table 1:** Percent injected activity in sentinel lymph node and in the contralateral lymph node (control) one hour after ^68^Ga-labelled SPION injection.

	Percentage injected activity (%) in SLN	Percentage injected activity (%) in control lymph node
Rat 1	4.08	0.13
Rat 2	0.97	0.08
Rat 3	1.14	0.16
Rat 4	0.41	0.04
Rat 5	7.92	0.11
Rat 6	0.80	0.15

### MMUS imaging

The six animals were divided in two groups, three animals in each. The sentinel lymph nodes of the animals in the first group were imaged with MMUS after two days post injection, while the animals in the second group were imaged after four days. A previously developed algorithm^21^ was employed to extract the magnetomotive displacement at each image pixel location in the imaged area. Magnetomotive displacement was detectable in four out of the six animals. Figure 1B shows a MMUS image of the same sentinel lymph node as in Fig. 1A where colour-coding with regard to the magnetomotive displacement is mapped onto the ultrasound B-mode image.

**Figure 2:**
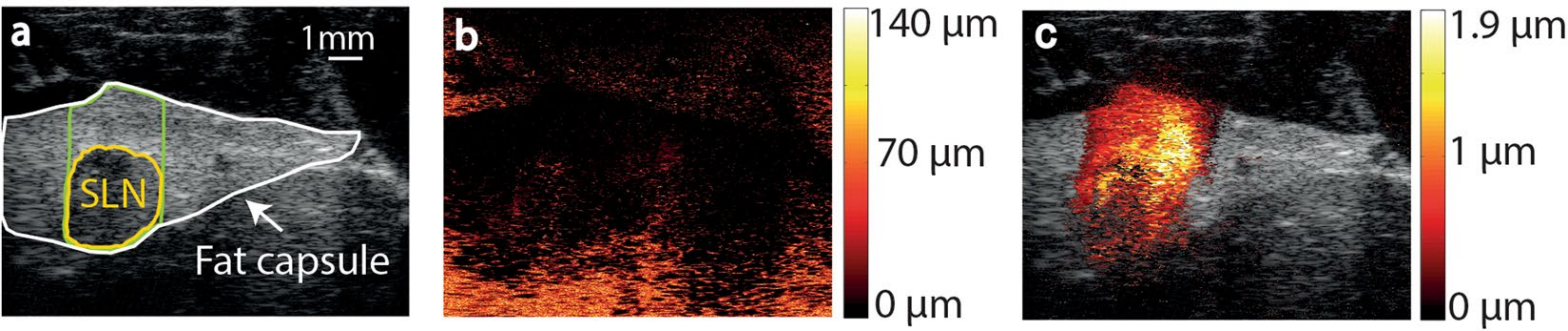
Images of the sentinel lymph node (SLN) and surrounding fat capsule in animal 5, obtained at an electromagnet excitation of 60 V at 5 Hz. (**a**) B-mode ultrasound image in which the SLN (yellow), the area where the magnetomotive ultrasound displacement was calculated (green), and the fat capsule (white) are outlined. (**b**) The total movement, i.e. the movement at all frequencies and phases, shown in colour-code for the area corresponding to that in the ultrasound image shown in panel A. The colour corresponds to displacement magnitude according to the colour-bar to the right. (**c**) MMUS image. In the image, displacement is only colour-coded when occurring with the same frequency and phase (± 0.35 radians) as the applied magnetic field. The colour-coded MMUS data is overlaid on the ultrasound B-mode image. Note that the maximum displacement magnitude is more than 70 times greater in panel (**b**) than panel (**c**).

The MMUS algorithm extracts the displacement occurring at precisely the frequency and phase of the applied magnetic field, thus suppressing all other artefactual motion. The efficacy of the algorithm is illustrated in a series of images in Fig. 2. Panel A shows a B-mode ultrasound image of a sentinel lymph node and its surrounding fat capsule. In panel B the total movement, i.e. the movement at all frequencies and with all phases, is overlaid as colour-code on the ultrasound B-mode image. Panel C shows the MMUS image. In this image, only pixels showing displacement with the same frequency and phase ( ± 0.35 radians) as the applied magnetic field are colour-coded and overlaid on the ultrasound B-mode image.

**Figure 3:**
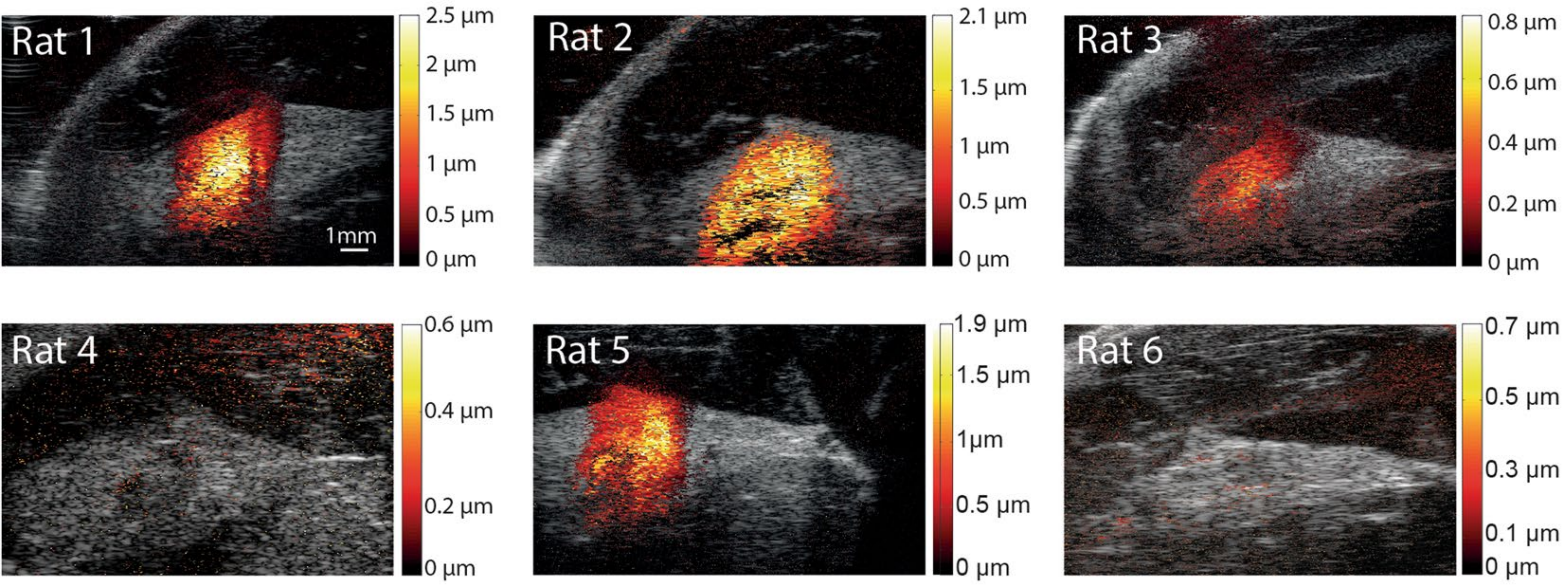
Magnetomotive ultrasound images of the sentinel lymp node and its surrounding fat capsule in all six animals. The images for animal 1, 2, 3 and 5 were obtained at an electromagnet exitation voltage of 40 V 5 Hz whereas the images for animal 4 and 6 were obtained at 60 V 5 Hz. The higher voltage setting is shown since no detectable magnetomotive displacement was found in animal 4 and 6. For animal 1, 2, and 5 bleeding of the magnetomotive signal outside the sentinel lymph node and fat capsule occurred in the MMUS images for the 60 V setting. This make the images difficult to compare, thus the 40 V setting is presented. The colour represents magnetomotive displacement according to the colour-bars to the right.

The MMUS images of the SLN in all six animals are shown in Fig. 3. The mean (± standard deviation) magnetomotive displacement in each SLN (displacement in there image cross sections and at six applied magnetic coil excitation voltages analyzed by there different observers) was 0.88 ± 0.09 µm for animal 1, 0.52 ± 0.01 µm for animal 2, 0.21 ± 0.01 µm for amimal 3, and 0.54 ± 0.03 µm for animal 5. No detectable magnetomotive displacement was found in animals 4 and 6 and thus no analysis of these two animals was performed.

### MR imaging

**Figure 4:**
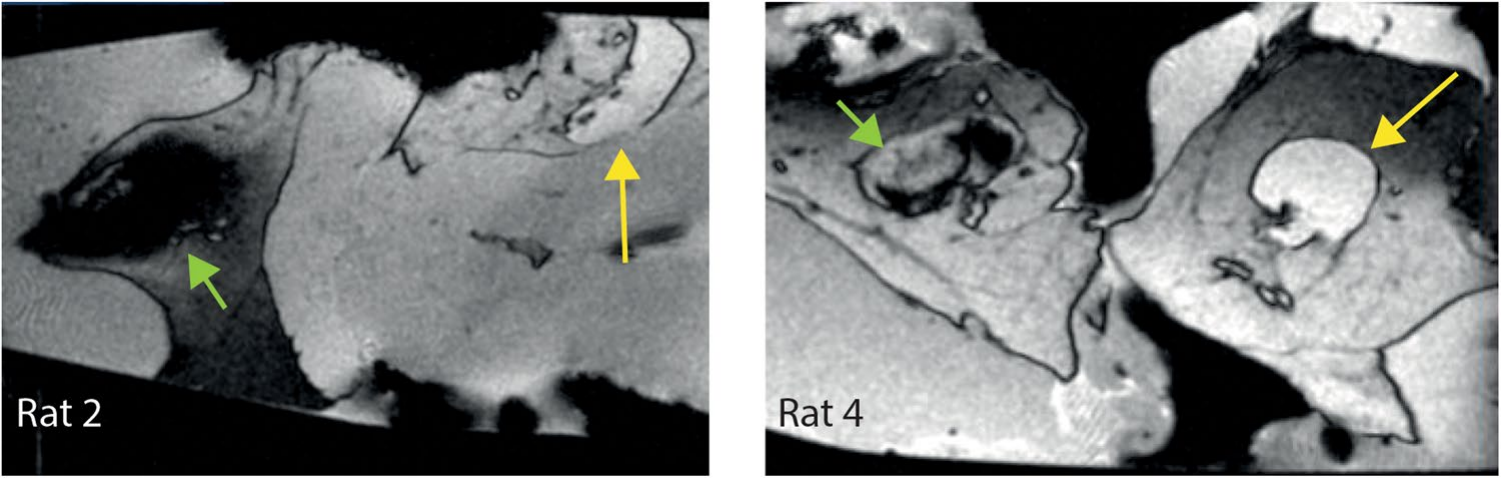
MR images of the excised tissues containing the sentinel lymph node (green shortshafted arrow) and control node (yellow long-shafted arrow) for animal 2 (detectable with MMUS) and animal 4 (not detectable with MMUS). Both lymph node samples (together with some of their surrounding tissue) were placed in an Eppendorf tube during the MRI acquisition. The superparamagnetic nanoparticles present in the sentinel lymph node are shown as hypoenhanced voids (black). The black areas around and in between the two tissue samples stem from air pockets. A considerably larger hypoenhancement can be seen for animal 2 compared to animal 4.

SPIONs lend themselves excellently as a contrast agent for MRI (and are indeed clinically approved as such^18, 28^, although not the very SPIONs used in this study) because of susceptibility effects from the iron core, which shows as hypoenhanced (dark) areas in the image where particles are present. The particles used in this study could therefore have served as a multimodal contrast agent for hybrid PET/MRI-systems. But the purpose of using MRI in this study, was only to make a semi-quantitative assessment of the amount of SPIONs accumulated in the sentinel lymph node for comparison with the MMUS signal. This was achieved by evaluating the extent of the hypoenhanced area in the MR images. For the four animals where MMUS signal was detected, a larger area of hypoenhancement could be seen in the MR images compared to animals without detectable MMUS signal (Fig. 4).

**Figure 5:**
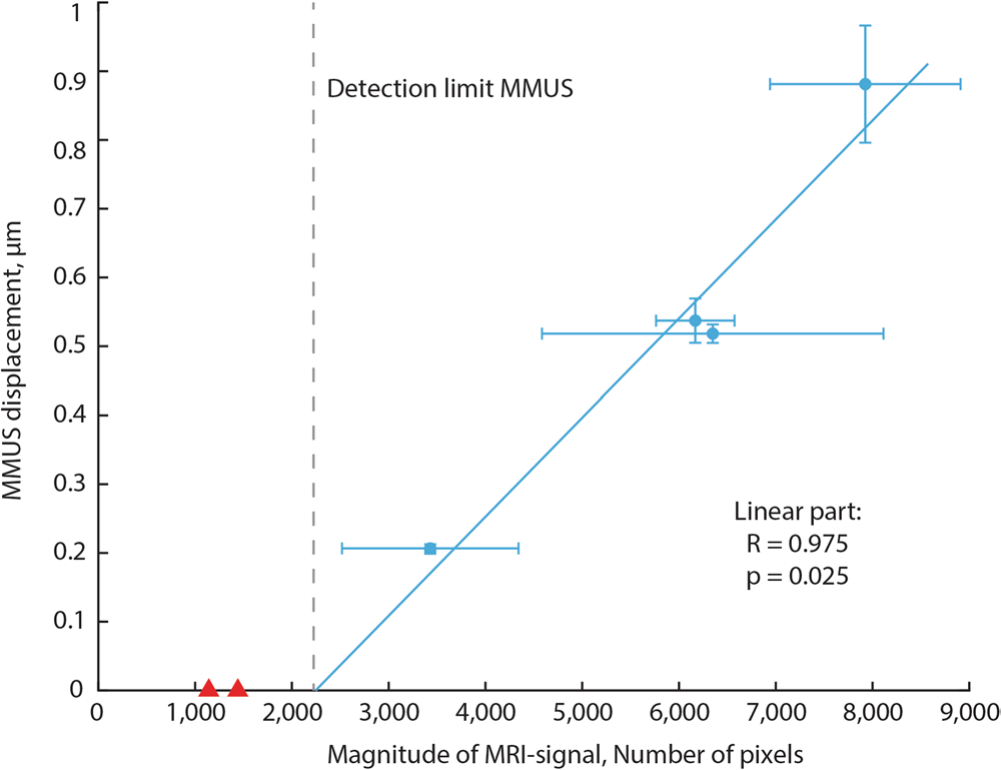
The solid line is a linear regression analysis of the relationship between the MMUS and MRI signals for the four rats with detectable MMUS signal. Correlation of the observations is evaluated using least squares regression analysis with calculation of Pearson’s product moment correlation coefficient, R statistics = 0.975 and p = 0.025. The error bars show the standard deviations of the mean for three observers, i.e. three mean values for each animal (each of these mean values are calculated from the 18 cross sections). The measurement values are displayed as asterisks (*). The two rats where no MMUS signal was found, are displayed as triangles (Δ) in the graph. An indication of the existence of a MMUS detection limit is presented as a dashed line in the graph.

When correlating the average magnetomotive displacement values in those four animals against the determined areas of hypoenhancement in the MR images in the same animals, a Pearson’s product moment correlation coefficient of 0.975 (p = 0.025, testing the hypothesis of no correlation) was obtained. This result is confined to the linear part as shown in Fig. 5. For the two animals where no MMUS-signal was detected the hypoenhanced regions were smaller than for the other four animals, indicated by triangles in Fig. 5.

## Discussion

We have shown the use for a multimodal imaging method in which PET/CT and MRI are combined with MMUS. To demonstrate the technique in a clinically relevant situation, we have imaged SLNs *in vivo* using ^68^Ga-labelled SPIONs in a rat model of lymphatic drainage. The combination of imaging modalities is a proof of concept for whole-body scanning of patients with hybrid PET/MRI systems, in which sensitive PET imaging and high-resolution MRI are followed by intra-operative MMUS several days later. The results indicated a strong correlation between MMUS and MRI measurements, while there seems to exist a threshold effect that limits the sensitivity for MMUS at lower SPION concentrations.

The SLN could clearly be identified in all six animals in the PET/CT images. After the SPION injection, the animals were awake for one hour before the PET scan was performed. Because lymph flow is to a large degree propelled by contraction of adjacent skeletal muscles, large variations in SPION accumulation in the SLN can be expected^29^, which was also confirmed here (Table 1). The mean activity uptake (± standard deviation) in the SLNs was found to be 2.6 ± 2.9% and this is in accordance with other published data^30^.

In the four animals where magnetomotive displacement could be detected, the results were strongly correlated with the results from the MRI scans (Figs 3–5) (Pearson’s product moment correlation coefficient = 0.975, p-value = 0.025) even though the low number of data points makes this more of an indicative correlation. The area of hypoenhancement in the MR images revealed that the concentration of SPIONs was lowest in the two animals in which no MMUS signal was observed. The concentration of nanoparticles in animals 4 and 6 was most probably not large enough to induce detectable magnetomotion in the nanoparticle-laden tissue. This kind of concentration threshold has been reported previously^21, 31^. We hypostasize that the main cause of this threshold is a consequence of internal mechanical resistance in tissue, which has to be exceeded before magnetomotive displacement can take place. In Fig. S1 in the supplementary data section the excitation voltage is plotted against the magnetomotive displacement for the four animals detectable with MMUS. The measurement data indicates that there is a threshold for animal three since extrapolation shows that a voltage of approximately 8 V is needed to induce magnetomotive displacement. In Fig. 5, the magnetomotive detection limit is denoted as a dashed line and also this figure indicates that a threshold exist. One possible way to lower the threshold value is to apply a stronger magnetic field and thereby increase the magnetic force acting on the nanoparticles (see animal 3 in Fig. S1 in supplementary data). Another way is to use nanoparticles with a higher magnetic susceptibility, e.g. zinc-doped iron oxide nanoparticles^24^.

The animals in the group injected four days before the MMUS examination (animals 1, 2, and 3) were all successfully imaged with MMUS, whereas only one animal (animal 5) in the group injected two days before could be imaged with MMUS. It can be argued that this is a result of the particles not having enough time to migrate to the SLN. However, from previous studies we have established that the period between injection and imaging in this study should be more than sufficient for SLN uptake^23, 29, 30, 32^. The reason for the variability within this group is instead most probably a result of animal 4 receiving the lowest concentration of SPIONs and a liver uptake of SPIONs in animal 6, likely due to the particles partly being injected into the bloodstream. The concentration of SPIONs in the injected volume was also highest for animals 1 and 2, which also affected the results.

As an intra-operative alternative to MMUS, other imaging methods such as optical imaging (e.g. optical coherence tomography (OCT)) and photoacoustic imaging should be mentioned. However, the penetration depth of these imaging methods is smaller, 1–2 mm (OCT)^33^ and a few centimeters, respectively^34^, than for ultrasound which makes their potential clinical applications more limited compared to MMUS imaging. Additionally, as demonstrated in this study, the MMUS technique has shown the potential to be used as a candidate for multimodal imaging together with PET and MRI. Magnetomotive OCT (MMOCT)^35^, based on the same key principle as MMUS, also possess this multimodal possibility^36^, but similar to OCT, the MMOCT technique suffers from poor penetration depth and is thus suitable for other imaging applications than MMUS.

To make the MMUS technique more suitable for future clinical use, a new probe design to facilitate the magnetic field excitation is necessary. Previously efforts to create such a design have been made by Pope *et al*.^19^ who have developed an open-air MMUS system where the magnetic field generator is applied from the same side as the ultrasound which is fundamental for clinical application, as well as extending the investigation depth (here limited to 10–15 mm, similar to the ultrasound penetration for the probe used). By labelling the nanoparticles with targeting molecules, e.g. antibodies or peptides, the particles might also be used for target-specific molecular imaging. A new array of applications in addition to lymph node imaging might thus be opened up for the MMUS technique.

## Material and Methods

### Nanoparticles

The SPIONs used in this study were obtained from Genovis AB (Lund, Sweden). The nanoparticles consisted of a Fe_3_O_4_ core (10 ± 2 nm) and were coated with polyethylene glycol to make the particles biocompatible and to prevent aggregation. Their hydrodynamic diameter was determined to 40 ± 5 nm by dynamic light scattering (Malvern Instruments Ltd, UK).

^68^Ga (T_1/2_ = 67.7 min, β +  = 89% and EC = 11%) was eluted from a ^68^Ga/^68^Ge-generator system (Eckert & Ziegler, Germany) with hydrochloric acid (6 mL of 0.6 M) in 0.3–0.5 mL fractions. Fractions containing 40–80 MBq of ^68^GaCl_3_ were used for labelling.

The ^68^Ga-labelling conditions and the radiolabelling were previously optimised by Madru *et al*.^30^. Briefly, the pH of the ^68^GaCl_3_ eluate was adjusted to pH 5.5 with NaOH (5–6 μL 5 M) and sodium acetate (100–200 µL of 0.25 M) before the SPIONs were added to the radionuclide solution. The reaction mixture was incubated in a heating box under gentle shaking conditions (50 °C for 25–30 min).

The ^68^Ga-labelled SPIONs were separated from free ^68^Ga using a column containing ferromagnetic spheres (Miltenyi Biotech, Germany). By attaching a magnet to the column and filtering the reaction mixture, ^68^Ga-labelled SPIONs were trapped within the column while free ^68^Ga and buffer solution flowed through. The magnet was removed and the ^68^Ga-SPIONs were eluted with saline. The labelling efficiency was 75–92%. The serum stability of the ^68^Ga-SPIONs has earlier been evaluated to be highly stable in human serum, with > 93% of radioactivity still attached to the SPIONs at 37 °C^30^.

To obtain equal injected activity between animals, SPIONs were labelled in four batches.

The activity of the injections was kept as constant as possible (4.4–8.0 MBq), which led to a variation in nanoparticle concentration (1–4 mg Fe/ml).

### Animals

The animal study was approved by the Local Ethics Committee for Animal Research, Lund, Sweden and was performed in compliance with local and national regulations.

Six 6-week-old female Wistar rats were subcutaneously injected with ^68^Ga-labelled SPION solution (0.1 mL) on the dorsal side of the right hind paw while under isoflurane anesthesia. The lymph node draining this injection site is the popliteal lymph node, which thereby acted as the SLN in our model. After injection, the anesthesia was removed and the animals were allowed to wake up and move around for one hour before the PET/CT scan was performed.

### PET/CT imaging

At one-hour post injection, each animal was anesthetized with isoflurane and placed on an animal bed (Minerve, Bioscan, USA) and imaged with a dual-modality PET/CT system (NanoPET/CT, Bioscan, USA). The injected paw was placed in a lead cylinder during image acquisition to shield the injection site from adding too many background counts to the SLN image. A five-minute CT scan was performed, followed by a PET scan in which three consecutive images were collected in 15 min intervals for 45 minutes. After the acquisition, the animals were returned to their cages.

Reconstruction of the images was performed using an ordered subset expectation maximum iterative method with the following parameters: energy window 400–600 keV, single slice rebinning to 2D LOR, a ring difference of 16 and a coincidence of 1–3. Morphological information was provided by CT images acquired with 55 keV peak, 360 projections, and medium or maximum zoom and were reconstructed with a RamLack filter. The PET and CT images were exported to VivoQuant (inviCRO, USA) for co-registration and analysis.

The SLN in each animal was delineated in the PET images by selecting a 3D ROI volume with the border at about 25% of the max activity concentration in the lymph node. The activity calculations of the ^68^Ga-labelled SPIONs were performed using a standard sample of ^68^Ga activity measured in the same geometry. In the contralateral leg, a volume of the same size as the one in the injected leg was delineated at the popliteal lymph node position and the same calculations were performed.

### MMUS imaging

**Figure 6:**
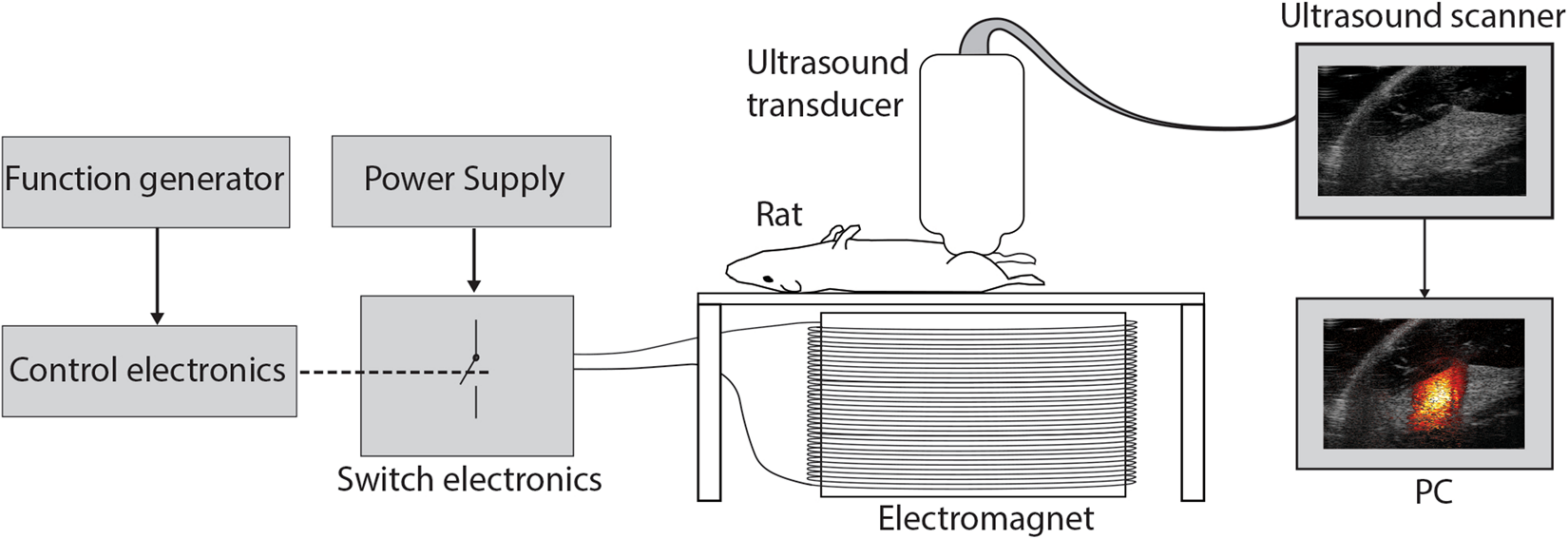
Magnetomotive ultrasound experimental setup. The lymph node in the animal’s knee was placed over the electromagnet. An electrical signal produced by a function generator was used to switch a power source at a frequency of 5 Hz, producing and a time-varying magnetic field of 5 Hz after application to the electromagnet. The nanoparticles in the sentinel lymph node were thereby set in motion and consequently their surrounding tissues. An ultrasound scanner registered the tissue movement, and post-signal processing of the ultrasound data extracted the magnetomotive displacement. The animal and the transducer were decoupled from the electromagnet to avoid mechanical disturbance from the electromagnet.

A key goal in this study was to determine if the amount of SPIONs in the SLN is still large enough to create a detectable magnetomotive displacement even days after SPION injection. A previous study shows that the uptake of SPIONs reaches a plateau at approximately 6 h post injection and that the amount of particles is not appreciably diminished after 72 h^29^. Therefore, we chose to divide the animals into two groups with three individuals in each with MMUS imaging being performed either two or four days after SPION administration. The imaging was performed with a high-frequency VisualSonics Vevo 2100 ultrasound scanner (VisualSonics Inc., Canada). The entire MMUS setup is illustrated in Fig. 6. Each animal was anesthetized with isoflurane, and its right knee was positioned between the ultrasound transducer (MS550D, centre frequency 32 MHz) and an electromagnet made from copper wire wound around an iron core made of structural steel. The core was designed to generate a uniform magnetic force at each depth across the image plane in the horizontal direction while the magnetic field decreased with distance from the iron core. This was achieved by making the iron core oblong, with the longer dimension exceeding the length of the ultrasound array. The animal and transducer were mechanically decoupled from the electromagnet to avoid mechanical vibrations from the electromagnet. An electrical signal produced by a function generator was used to switch a power source at a frequency of 5 Hz, thus forming a 5 Hz square wave with a 50% duty cycle. The voltage setting on the power source was varied (from 10 V_peak-to-peak_ to 60 V_peak-to-peak_ in 10 V increments, switching the voltage between 0 V and the peak voltage) to produce six magnitudes of the magnetic field after application to the electromagnet, since responses vary with voltage in different animals (see Supplementary Fig. S1). At a distance of one centimeter from the iron core, the approximately position of the SLNs during MMUS imaging, the magnetic flux density was measured to be 0.33 T and the field gradient 24 T/m (peak values) at the highest voltage setting (60 V). The mean power used was varied between 51 W and 2.0 kW (10 V and 60 V, respectively). At each power level, three different cross sections were obtained for each animal, except from animal 5 where only two cross sections were collected due to a technical problem. The signal generated by the function generator, i.e. the reference signal, was fed into the ultrasound system via the ECG input, and this enabled acquisition of synchronized measurements. For each measurement, ultrasound data were collected for three seconds at a recording rate of 50 frames per second. The ultrasound data were post-processed with our own in-house developed MMUS algorithm implemented in Matlab (The MathWorks Inc., USA)^21^.

### Frequency and phase tracking MMUS algorithm

The purpose of the algorithm was to isolate the MMUS signal from motion artifacts caused by for example heartbeats and respiration, which had much higher magnitudes. Because the particles move in compliance with the magnetic field, motion at 5 Hz was tracked by the algorithm in the ultrasound speckle echoes over time. All other frequencies were suppressed and filtered out. Discrimination with regard to the phase of the nanoparticles was also performed, and only pixels showing movement with the same phase (± 0.35 radians) as the excitation signal were accepted as nanoparticle movement. For a more detailed description of the algorithm see reference^21^.

The popliteal lymph nodes are situated in the knee inside a fat capsule. At higher excitation voltages, the generated magnetomotion can be observed outside the SLN in the surrounding fatty tissue^23, 32^. We chose to include all of this motion in the evaluation of the magnetomotive displacement signal. In previous studies^21, 23, 32, 37^, we showed that magnetomotion propagates from the area containing SPIONs, but it can also be the result of magnetomotion generated outside the imaging plane. In either case, the detected magnetomotion reflects the total deposited energy, which motivates us to also include magnetomotion that occurs vertically inside the fat capsule. Thus, the SLN as well as the area of the fat capsule extending above and below the SLN was outlined in the ultrasound B-mode grayscale images (outlined in green in Fig. 4). For each pixel in the outlined area, the magnetomotive movement, i.e. the movement at the precise frequency and phase (± 0.35 radians) as the nanoparticles, was calculated using our algorithm. To obtain a value to represent the magnetomotive movement in each SLN, the average magnetomotive movement of all pixels in the outlined area was calculated. Finally, to obtain a value to represent the magnetomotive movement in each animal, the average magnetomotive movement of all cross sections (3 per SLN) and at all voltages (6 per cross section) in that specific animal was calculated. Thus the presented value of the magnetomotive movement for each animal, was obtained from 18 individual images of that animal (except for animal 5 where only two cross sections were collected). Three different observers performed these segmentations and calculations in all animals to avoid observer bias. A mean of the three observers’ results was calculated to represent the magnetomotive displacement in each animal.

After the MMUS examination, the animals were sacrificed by an isoflurane overdose. The SLN and the popliteal lymph node from the contralateral side were dissected together with their surrounding tissue for further MRI analysis.

### *Ex-vivo* MR imaging

An 11.7 T vertical wide-bore MR scanner (Agilent Technologies) was used to image the excised lymph nodes using a micro-imaging RF coil (13 mm diameter, Neos Biotec, Spain). For all six animals, both the SLN and control node, still encapsulated in some of the surrounding muscle and fat, were put in an Eppendorf tube that was placed inside the MRI scanner. T2*-weighted MR images were acquired (3D gradient echo, TE 1.48 ms, TR 15 ms, field of view: 20 × 10 × 10 mm, pixel resolution after zero filling: 39 × 39 × 39 μm, 4 averages, and 16 min scan time).

The 3D dataset containing 256 MR images of 512 × 256 voxels was exported to ImageJ^38^. The principal contrast effect from SPIO particles originates from a strong T2*-relaxation due to susceptibility effects caused by the iron oxide core. This shows as hypoenhancement in the MR images. To evaluate the MR images, the hypointense MRI signal in the lymphatic tissue was quantified under the assumption that the number of black pixels in the hypoenhancement was proportional to the amount of SPIONs. The cross section with the highest effect on the MR signal was located in the 3D volume, and an image of this cross section was saved. The black pixels in the SLN, and sometimes also in its closest surrounding tissue (depending on signal extent), were outlined in Adobe Photoshop (Adobe Systems, USA) using the magic wand tool. The number of selected pixels was then counted using a Matlab script. In the same manner as for the MMUS measurements, three observers analyzed the MRI data to avoid operator bias and a mean value for each animal was calculated.

The relation between MMUS displacement and magnitude of the MRI-signal was evaluated using least squares regression analysis with calculation of Pearson’s product moment correlation coefficient.

## Electronic supplementary material

Supplementary Information
