# Supplementary material for: Combined Magnetomotive ultrasound, PET/CT, and MR imaging of 68Ga-labelled superparamagnetic iron oxide nanoparticles in rat sentinel lymph nodes in vivo
Source: Sci Rep. 2017 Jul 6;7:4824. doi: 10.1038/s41598-017-04396-z (PMC5500498; doi:10.1038/s41598-017-04396-z)
Supplement: Supplementary file 1 — Supplementary Information [file 41598_2017_4396_MOESM1_ESM.pdf]

Combined Magnetomotive ultrasound, PET/CT, and MR imaging of ^{68}Ga-labelled superparamagnetic iron oxide nanoparticles in rat sentinel lymph nodes *in vivo*

Maria Evertsson, Pontus Kjellman, Magnus Cinthio, Roger Andersson, Thuy A Tran, Rene in 't Zandt, Gustav Grafström, Hanna Toftevall, Sarah Fredriksson, Christian Ingvar, Sven-Erik Strand, Tomas Jansson

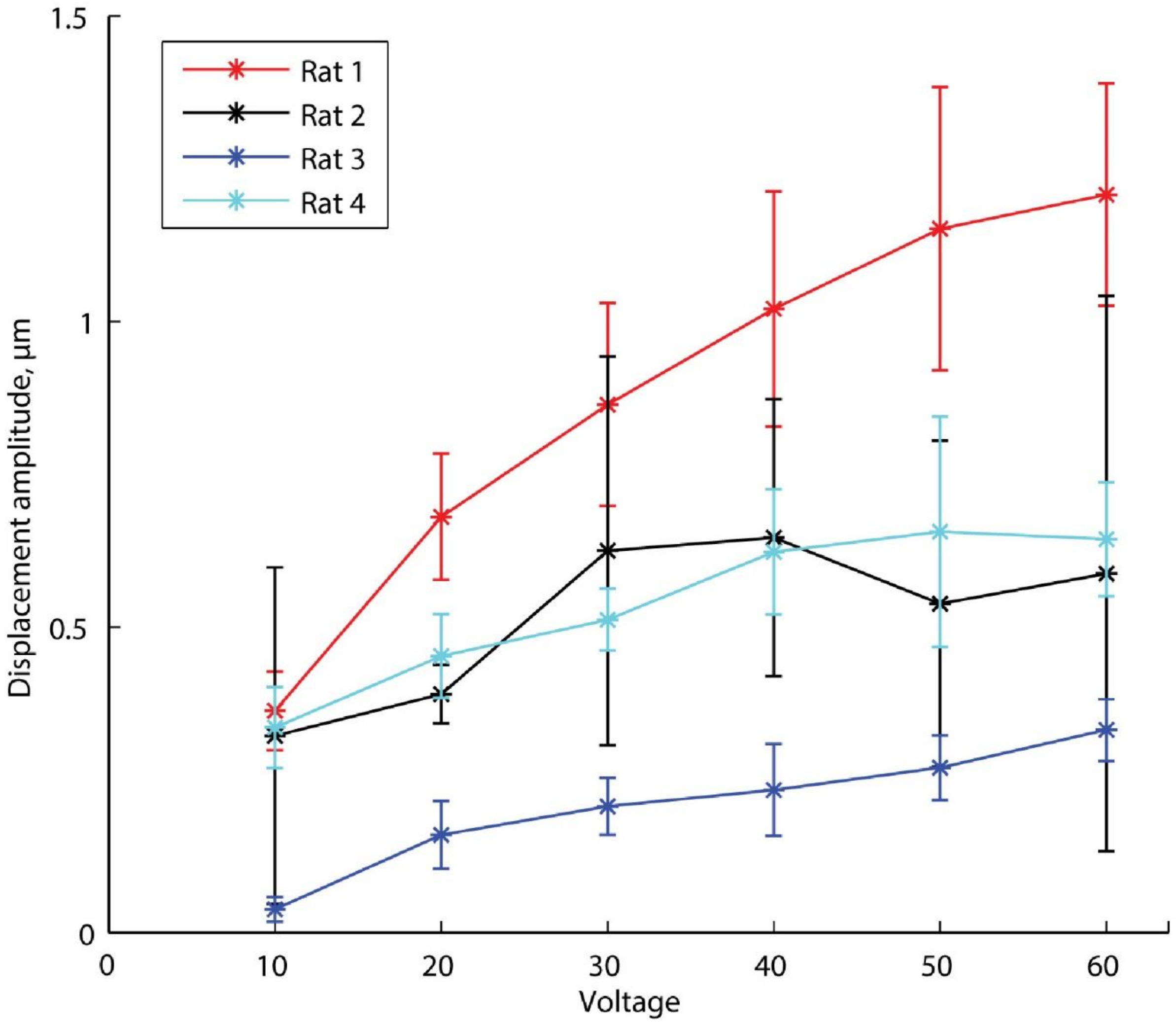

SUPPLEMENTAY FIGURE 1S. The graph shows the mean magnetomotive displacement in the lymph node for the four rats detectable with MMUS. For each rat and voltage three cross sections were obtained (Rat 1-3, rat 5 only two cross section due to a technical problem). The mean magnetomotive displacement in the lymph node area was calculated for each cross section at each voltage. Then a mean value of the magnetomotive displacement in each animal was calculated using all cross sections at all voltages. Three observers analysed the data, and each point in the graph represent the mean of these three observers and the error bars display the standard deviation of the mean for the three observers. The graph indicates that there seems to be a threshold voltage for Rat 3, since linear extrapolation suggests that approximately 8 V is needed before a detectable magnetomotive displacement can be induced. For the other three rats no such threshold value can be seen when the obtained measurement data was extrapolated linearly. The MRI revealed that the sentinel lymph node in Rat 3 contained the lowest concentration of SPIONs of the four rats and a lower magnetomotive displacement was thus to expect.
